# Supplementary material for: Transcriptome Analysis Revealed That Hydrogen Peroxide-Regulated Oxidative Phosphorylation Plays an Important Role in the Formation of Pleurotus ostreatus Cap Color
Source: J Fungi (Basel). 2023 Aug 3;9(8):823. doi: 10.3390/jof9080823 (PMC10455351; doi:10.3390/jof9080823)
Supplement: Supplementary file 1 [file jof-09-00823-s001.zip › jof-2478026-supplementary.pdf]

Table S1 Primers used in this study.

Primer	Sequence (5′→3′)	Note
qPCR_*g400*-F	CCATTCCACGACCAGACATC	
qPCR_*g400*-R	TTCAACGGAGTCCAAGCTATC	Complex I
qPCR_*g8839*-F	GGAGGAGCTTGGTACTTGAAG	
qPCR_*g8839*-R	ATGGTGTAGGGTTGGTCTTG	
qPCR_*g2860*-F	AGAGAACAGTGCCAAACAGG	
qPCR_*g2860*-R	GCAAGCATCCACGCAATG	
qPCR_*g2546*-F	TGCTACGTGGTATGCCAAC	Complex III
qPCR_*g2546*-R	GCTTGATGCGGAAAATACGG	
qPCR_*g13251*-F	ACGGTCTTGTGAAGCAGAG	
qPCR_*g13251*-R	GGAAAATATGGCGAGTTGGG	
qPCR_*g7640*-F	TCAAGGAGAAAGTGGAGAATGTC	
qPCR_*g7640*-R	CTCCTTCAAGTTGACACCGAG	
qPCR_*g12499*-F	CCAAAGCCGACCCTATCAAG	
qPCR_*g12499*-R	GGTGTAGACTGAGCCACATTC	
qPCR_*g12052*-F	GCTTCCAGAACTATACCGACTAC	
qPCR_*g12052*-R	TCCCACTTGCTGATCCATTC	
qPCR_*g8872*-F	GCGTCAGATCAATTCACACC	Complex IV
qPCR_*g8872*-R	GGGAATGCTGAAGCCAAATAAC	
qPCR_*g9081*-F	CTAACCCCATTGTACGCCG	
qPCR_*g9081*-R	CTAATCGTCCACCCATCAGAG	
qPCR_*g2230*-F	GGAAAATTTAGCGTTCTGGTCG	
qPCR_*g2230*-R	CTGACTTGGTCTGAGGACATG	
qPCR_*g9180*-F	TCCTCTCCAATACCTCGCTC	
qPCR_*g9180*-R	GACATTTGACGGTAAGCAACG	
qPCR_*g11242*-F	TCGTGGTATTGCTGAACTCG	
qPCR_*g11242*-R	GGAGGGACTTGTAATCTTGGAG	
qPCR_*g1602*-F	AAGTCCGTCAGAAACATCGAG	
qPCR_*g1602*-R	GTCAGTCGCAACCTTGAAAAC	
qPCR_*g12390*-F	CTTGTCAGAGAACGGTCGG	
qPCR_*g12390*-R	GGGTGAGAATATCCTTGGGC	Complex V
qPCR_*g10165*-F	GGTCTCTTTGCTCTCTCCAAG	
qPCR_*g10165*-R	TGTTCGCTCTCCTTCAACTG	
qPCR_*g8845*-F	TTTCAAGCCTGTCACCTACG	
qPCR_*g8845*-R	TCCTCGATGTTAGCCAAAGTC	
qPCR_*g12225*-F	ACATCCAAAAGCAAACCCATG	
qPCR_*g12225*-R	GCTTCTCTAGATCGAAACGAGG	
qPCR_*g11401*-F	TCCCAAAATCGCTTCTCCG	

qPCR_*g11401*-R	GCATTGCTACCGTTGAAGAAC	
qPCR_*g12829*-F	CGCTTACTCAACACTTTGGTC	
qPCR_*g12829*-R	TTCGTCGGCCAATAATCTCC	
qPCR_*g8493*-F	CTGGAAGCAACTGTAAAATGGC	
qPCR_*g8493*-R	CAAACTCTGGTGATCGGACAG	
qPCR_*g3195*-F	CTTCTCTACCATTGGCGTCG	
qPCR_*g3195*-R	CCTGATAAACTGCTCTTCCTCG	
qPCR_*g10989*-F	GACATTCAGACCCGAGCTAATC	
qPCR_*g10989*-R	AATCCAGCAAAGAGCGAGTAG	
qPCR_*tubulin*-F	AGGCTTTCTTGCATTGGTACACGC	References
qPCR_*tubulin*-R	TATTCGCCTTCTTCCTCATCGGCA	
qPCR_*actin*-F	AGTCGGTGCCTTGGTTAT	
qPCR_*actin*-R	ATACCGACCATCACACCT	
